# Key Genes Are Associated with the Prognosis of Glioma, and Melittin Can Regulate the Expression of These Genes in Glioma U87 Cells

**DOI:** 10.1155/2022/7033478

**Published:** 2022-12-31

**Authors:** Ran Li, Ting Tao, Qiuyun Ren, Sujun Xie, Xiaofen Gao, Jie Wu, Diling Chen, Changqiong Xu

**Affiliations:** ^1^Guangdong Provincial Key Laboratory of Utilization and Conservation of Food and Medicinal Resources in Northern Region, Shaoguan University, 288 Daxue Road, Shaoguan, 512005 Guangdong Province, China; ^2^Medical College of Shaoguan University, 108 XinHua Nan Road, Shaoguan, 512005 Guangdong Province, China; ^3^Hunan Yueyang Maternal & Child Health-Care Hospital, 693 Baling Middle Road, Yueyang, 414000 Hunan Province, China; ^4^Brain Function and Disease Laboratory, Shantou University Medical College, 22 Xinling Road, Shantou, 515041 Guangdong Province, China; ^5^Guangzhou University of Chinese Medicine, 12 Jichang Road, Baiyun District, Guangzhou, 510405 Guangdong Province, China; ^6^Guangzhou Laboratory, 9 XingDao HuanBei Road, Guangzhou International Bio Island, Guangzhou, 510005 Guangdong Province, China

## Abstract

Glioma is the most common primary tumor of the central nervous system. Currently, there is no effective treatment for glioma. Melittin (MT) is the main component of bee venom, which was found to have therapeutic effects on a variety of tumors. In this study, we explored the relationship between key genes regulated by MT and the prognosis of glioma. In cultured glioma U87 and U251 cells, MT inhibited cell proliferation and induces cell apoptosis in a time- and concentration-dependent manner. RNA-seq revealed that MT upregulated 11 genes and downregulated 37 genes. These genes are mainly enriched in cell membrane signaling pathways, such as surface membrane, membrane-enclosed organelles, integral component of membrane, PPAR signaling pathway, and voltage-gated potassium channel. PPI network analysis and literature analysis of 48 genes were performed, and 8 key genes were identified, and these key genes were closely associated with clinical prognosis. Overexpression of PCDH18, PPL, DEPP1, VASN, KCNE4, MYBPH, and C5AR2 genes or low expression of MARCH4 gene in glioma patients was associated with poor survival. qPCR confirmed that MT can regulate the expression of these genes in glioma U87 cells. This study indicated that MT significantly inhibited the growth and regulated the expression of PCDH18, C5AR2, VASN, DEPP1, MYBPH, KCNE4, PPL, and MARCH4 genes in glioma U87 cells in vitro. These genes are closely related to the prognosis of patients with glioma and can be used as independent prognostic factors in patients with glioma. MT is a potential drug for the treatment of glioma.

## 1. Introductions

Glioma is the most common primary malignant tumor of the brain. It has high morbidity and low survival rate [1–3]. In the 2021 World Health Organization (WHO) classification of central nervous system (CNS) tumors, low-grade glioma (LGG) includes CNS WHO grade 1-2, and high-grade glioma (HGG) includes CNS WHO grade 3-4. LGG accounts for 6% of primary central nervous system tumors in adults and has a good prognosis [4]. However, compared with other benign intracranial tumors, there is still a higher recurrence rate. Glioblastoma multiforme (GBM) is WHO grade 4, which is the most aggressive and malignant primary brain tumor. Surgical is an important treatment for LGG. However, due to the aggressive growth of glioma, surgical treatment alone cannot completely cure the diffuse growth of the tumor. At present, adjuvant therapies such as radiotherapy and chemotherapy are mostly used to delay the tumor recurrence time. But LGG are still difficult to cure completely. For HGG, the current standard treatment includes surgical removal of the tumor [5], tumor-treating fields (TT Fields) [6], targeted therapy [7], immunotherapy [8], radiotherapy (RT) [9], and chemotherapy [10]. Despite the continuous updates and advances in diagnostic and therapeutic techniques, the prognosis of patients is not satisfactory. In recent years, TMZ has made some progress as the main drug in chemotherapy, but the prognosis of patients is still poor [11]. It is urgent to find effective treatment methods. Therefore, searching for effective treatment is still a hot spot in recent years.

**Figure 1:**
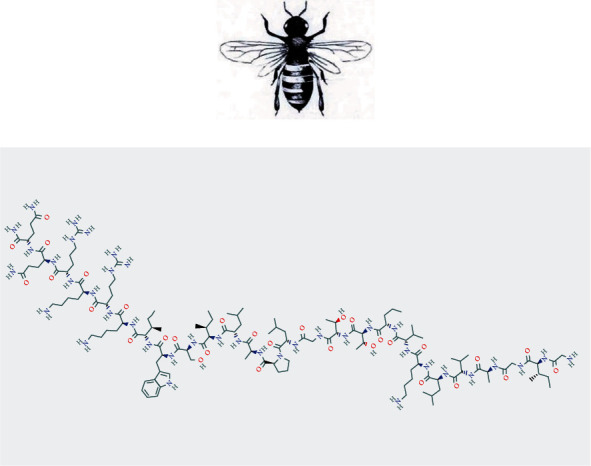
The honeybee and structure of MT (a, b).

MT is the main active component of honeybee venom, honeybee venom is a kind of fragrant transparent venom secreted by worker bee venom glands and accessory glands (Figure 1(a)). Among the many components of honeybee venom, the content of MT is the highest which makes up more than 40% of honeybee venom, MT is a polypeptide composed of 26 amino acid residues [12] (Figure 1(b)), the molecular formula is C131H2 29N39O31, and the relative molecular weight is 2846.46.

In recent years, there have been many studies on its pharmacological action and mechanisms. It has been reported that MT has antioxidant [13], antifungal [14], and anti-inflammatory [15, 16] pharmacological effects. Therefore, MT was used in the treatment of a variety of diseases, especially rheumatoid arthritis [17]. The mechanism may be related to regulating Th17/Treg balance [18] and inhibiting the activation of NF-*κ*B, STAT3, and bcl-2 expression induced by IL-6/S IL-6R complex [19]. In addition, previous studies have shown that MT destroys the cell membrane and leads to cell death. It does not need to enter the cells, but can show cytotoxicity to destroy tumor cells outside the cells [20]. MT is believed to have a proapoptotic effect and antitumor activity [21] by inhibition of the HIF-1 *α*/Akt pathway in liver cancer [22], NF-*κ*B signaling pathway in lung carcinoma cells [23], TGF-*β*-mediated ERK signaling pathway in lung cancer [24], Her2 enrichment and growth factor receptor activation in triple negative breast cancer [25], MAPK in melanoma [26], and JAK2/STAT3 in ovarian cancer [27]. In addition, some studies [28–30] have shown that MT inhibits proliferation and induces apoptosis of malignant human glioma cells. The majority of the antineoplastic activity of honeybee venom has been attributed to melittin through inhibition of VEGF, FLT-1, and MMP-9 in glioma C6 cells [31]; metaloprotease-2 in human glioblastoma cells [32]; and STAT3 and VEGF in glioma SHG44 cell [33]. However, information regarding the functional role of MT in glioma is limited. The targets and mechanism of action of MT in the treatment of glioma need to be further studied.

Therefore, on the basis of the previous studies, this study further explored the effect of MT on glioma. In this study, we evaluated the potential of MT in inhibiting U87 and U251 cells in vitro. We generated deep sequencing RNA data from U87- and MT-treated U87 cell samples, monitoring the differentially expressed genes. TCGA database was used to further screen genes related to the survival prognosis of glioma. We found that MT can regulate the expression of these genes associated with glioma prognosis in glioma U87 cells. That could be helpful for determining the molecular mechanism of MT in the treatment of glioma.

## 2. Methods

The methods were followed as previously described [34] with some modifications.

### 2.1. MT Preparation

MT (20449-79-0, Shanghai Baishun Biological Technology Co., Ltd., China) was dissolved in saline and stocked at -20°C.

### 2.2. Cell Culture

U251 and U87 cells were derived from glioblastoma tissue of patients. U87 cells were cultured from a grade III astrocytoma-glioblastoma of a 44-year-old woman. U251 cells were cultured from a 75-year-old patient. U87 cells (provided by professor Yi Guan) and U251 cells (Suzhou Institute of Cell Biology, Chinese Academy of Sciences) were cultured in Dulbecco modified Eagle medium (Gibco, USA) with 10% FBS (Gibco, USA) and 1% of penicillin-streptomycin at 37°C, in humidified air containing 5% of CO_2_.

### 2.3. Cell Viability

Cell proliferation was determined by using a Cell Counting Kit-8 (CCK-8, Meilunbio, Dalian, China). Cells were seeded into a 96-well plate (2 × 10^3^ cells/well) with 100 *μ*L of culture medium. After 12 h, cells were treated with various concentrations of MT (0, 30.0, 60.0, 150.0, 300.0, 600.0, and 1500.0 nM) for 24 and 48 h. 10 *μ*L CCK-8 solution was added to each well and cells were incubated at 37°C for 2 h. Light absorbance was measured at 450 nm using a microplate reader (Bio Tek Elx800, USA).

The cell growth inhibition and the median inhibitory concentration (IC_50_) were calculated from the cytotoxicity curves. Cell morphological changes were observed using an inverted microscope (Nikon, ECLIPSE E600, Tokyo, JPN).

### 2.4. Annexin V-FITC/PI Cell Apoptosis Analysis

Apoptosis was detected by flow cytometry via the examination of altered plasma membrane phospholipid packing by lipophilic dye Annexin V. Briefly, treated cells (treated with MT, 0-150.0 nM) were harvested by trypsin, washed twice with PBS, and then, resuspended in binding buffer. Thereafter, 5 *μ*L of Annexin V-FITC was added into cell suspension and incubated for 15 min at room temperature in the dark. PI staining was performed 5 min before evaluation by flow cytometry within 30 min (BD Accuri™ C6, BD Biosciences, San Jose, CA). Data analysis was performed with BD Accuri C6 software (Version 1.0.264.21).

### 2.5. Total RNA Isolation and Transcriptome Sequencing

U87 cells were seeded in a 6-well plate (1 × 10^6^ cells/well). After 12 h, cells were treated with MT (0 *μ*M and 150 nM for 12 h). Total RNA was isolated using a RNA extraction kit (Beyotime, Shanghai, China). Nanodrop 2000 spectrophotometer (Implen, Los Angeles, USA) was used to test RNA purity and concentration. After that, mRNA was enriched, the enriched mRNA was fragmented into short fragments and reverse transcribed into cDNA. Second-strand cDNA was synthesized by DNA polymerase I, RNase H, dNTP, and buffer. Then, the cDNA fragments were purified with a QIAQuick PCR extraction kit, end-repaired, poly(A) tailed, and ligated to Illumina sequencing adapters. The ligation products were size selected by agarose gel electrophoresis, PCR amplified, and sequenced using an Illumina HiSeqTM 2500 platform by Gene Denovo Biotechnology Co. (Guangzhou, China).

### 2.6. PPI Network Analysis and Key Genes

PPI network analysis plays a major role in predicting the functionality of interacting genes or proteins and gives an insight into the functional relationships and evolutionary conservation of interactions among the genes. An interaction network is a graphical representation of gene/protein interactome, where each gene/protein is a node, and interaction between gene/protein is an edge [35]. PPI was constructed using the String Database (https://string-db.org/), and the key genes were obtained.

### 2.7. Correlation Analysis between Key Genes and Survival Prognosis

Survival analysis [36] is a statistical method used to analyze the relationship between survival status and corresponding time. It has been widely used in medicine. Survival Rate represents the probability that the survival time of the patient is greater than time *t*, denoted by *R*(*t*). Time represents the actual survival time of the patient. The survival rate curve can be obtained if the horizontal axis is taken as the following prevention time *t* and the vertical axis is taken as the survival probability *R*(*t*).

Independent prognostic analysis of genes associated with glioma survival was performed to obtain independent prognostic factors associated with glioma. These genes as independent prognostic factors are of great clinical interest which suggested that these genes could be independent of other clinical traits as independent prognostic factors [37].

To further verify the relationship between the key genes and the survival prognosis of patients with glioma, RNA-seq data and clinical data of glioma were taken from The Cancer Genome Atlas (TCGA) database (https://tcga-data.nci.nih.gov/tcga/). According to the expression of DEG mRNA in glioma, DEGs were divided into high-expression group (above average) and low-expression group (below average), and the survival rate of patients was analyzed. Kaplan-Meier curve was used to draw the survival curve to detect the relationship between the expression level of DEGs and the survival of glioma patients. We then assessed the DEGs based on other clinical factors in the univariate and multivariate Cox proportional-hazard analysis.

### 2.8. Quantitative Real-Time PCR Analysis

Total RNA was isolated for RNA-seq. Based on the RNA-seq results, relevant genes were selected, and primers were designed using Primer Premier 5 software (primer sequences are shown in Table 1). Reverse transcription into cDNA was performed using a cDNA Synthesis SuperMix for qPCR Kit (Transgen, Beijing, China), and the cDNA was amplified using an RT-PCR amplification kit (PerfectStart Green qPCR SuperMix) (Transgen, Beijing, China). The 2^−ΔΔCt^ method was used to perform analysis with GAPDH as the reference gene.

### 2.9. Statistical Analysis

Data were analyzed using GraphPad Prism 7.0 (GraphPad Software, Inc. La Jolla, CA, USA) and SPSS software (v.24.0). Data are presented as mean ± standard error (SEM). Statistical analyses of multiple group comparisons were performed by two-way ANOVA (CCK8 analysis, IC_50_, apoptotic rate, qPCR). Clinical correlation analysis was performed using R (version 4.0.4, http://www.r-project.org). Cumulative survival time was calculated using the Kaplan-Meier method, and the differences in survival curves were analyzed using the log-rank test from “survival” package [38] (version: 2.41.3). Univariate and multivariate analyses were conducted using the Cox proportional hazard regression model. For all tests, significance was defined at ^∗^*p* < 0.05, ^∗∗^*p* < 0.01, ^∗∗∗^*p* < 0.001, and ^∗∗∗∗^*p* < 0.0001.

## 3. Results

### 3.1. MT Affects Cell Morphology and Suppresses Cell Proliferation

**Figure 2:**
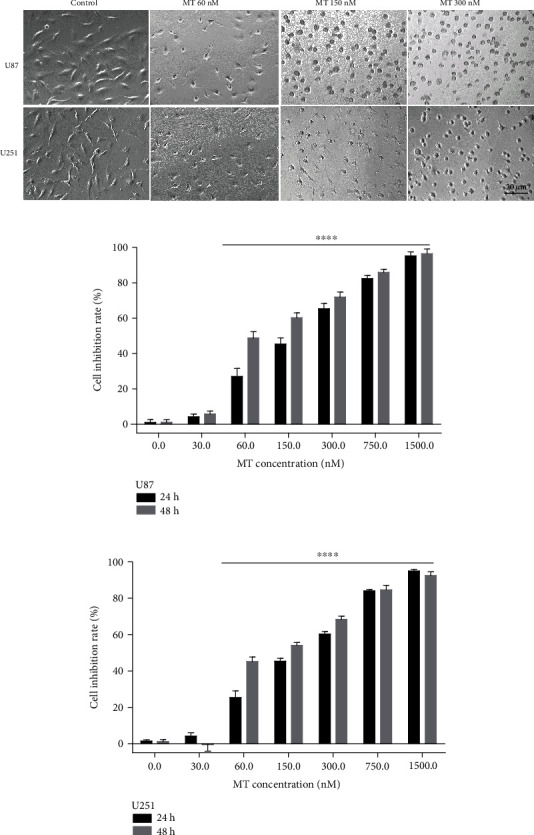
The effects of MT on cell growth of U87 and U251 glioma cells. (a) Morphological and cell density changes of U87 and U251 cells observed after 48 h treatment with different concentrations of MT. (b) Effects of MT on U87 cell proliferation using a CCK-8 assay. (c) Effects of MT on U251 cell proliferation using a CCK-8 assay. Data are presented as the mean ± SEM, *n* = 4, ^∗∗∗∗^*p* < 0.0001 vs. control group.

The human U87 and U251 cells were used to evaluate the pharmacological effects of MT. Cells were treated with different concentrations of MT for 48 h, then the cell morphological changes were examined. At the concentration of 60.0 nM group, cells exhibited morphological changes, such as cell rounding and the disappearance of protruding spike. At the concentration of 150 nM, treated cells showed cell shrinking, disappearance of spikes, and detachment from the substrate (Figure 2(a)).

CCK-8 assay on cell viability showed that MT treatment remarkably inhibited U87 cell growth in a concentration-dependent manner. The IC_50_ values of MT-induced inhibition at 24 and 48 h were 178.07 ± 6.67 and 125.71 ± 2.68 nM, respectively. In U251 cells, the IC_50_ values at 24 and 48 h were 185.20 ± 4.79 and 151.59 ± 10.79 nM, respectively (Figures 2(b) and 2(c), Table 2).

### 3.2. MT Induces Cell Apoptosis

**Figure 3:**
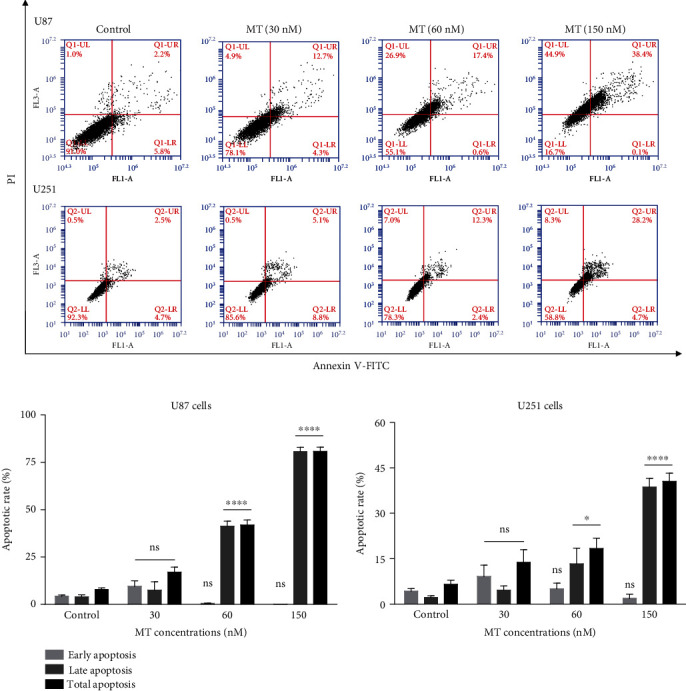
The effect of MT on apoptosis of U87 and U251 glioma cells. (a) Cells treated with different concentrations of MT for 48 h. Cell apoptosis detected by flow cytometry. The lower left represents normal cells, the lower right represents early apoptotic cells, the upper right represents late apoptotic cells, and the upper left represents dead or late apoptotic cells. (b) Analysis of the altered rate of early apoptosis, late apoptosis, and total apoptosis after MT treatment. Results are expressed as means ± SEM, *n* = 3, ^∗∗∗∗^*p* < 0.0001 vs. the control group.

To evaluate the effects of MT on cell apoptosis, U87 and U251 cells were treated with different concentrations of MT, then examined for cell apoptosis by Annexin V-FITC/PI flow cytometry (Figure 3(a)). After treatment with 30.0, 60.0, and 150.0 nM MT on U87 cells, the early apoptotic rate increased from 4.27 ± 0.72% (untreated control group) to 9.57 ± 2.91%, then decreased to 0.57 ± 0.03% and 0.10% ± 0.00%, respectively. The decrease of early apoptosis at 60.0 and 150.0 nM MT may be due to the rapid occurrence of late apoptosis and even death of tumor cells. Late apoptotic rate increased from 3.93 ± 1.10% (control) to 7.50 ± 4.38%, 41.30 ± 2.64%, and 80.70 ± 2.25% at 30.0, 60.0, and 150.0 nM MT, respectively. Thus, the total apoptotic rate increased from 7.87 ± 0.75% (control) to 17.07 ± 2.59%, 41.87 ± 2.67%, and 80.80 ± 2.25% at 30.0, 60.0, and 150.0 nM MT, respectively. There was a significant difference between the 60.0 and 150.0 nM (^∗∗∗∗^*p* < 0.0001 vs. control) in late and total apoptosis. In U251 cells, we observed that MT had a statistically significant effect on the late apoptosis and total apoptosis of U251 cells at 60 nM (^∗^*p* < 0.05 vs. control), while MT had a statistically significant effect on the late apoptosis and total apoptosis of U251 cells at 150 nM (^∗∗∗∗^*p* < 0.0001 vs. control) (Figure 3(b)).

### 3.3. Transcriptome Sequencing

**Figure 4:**
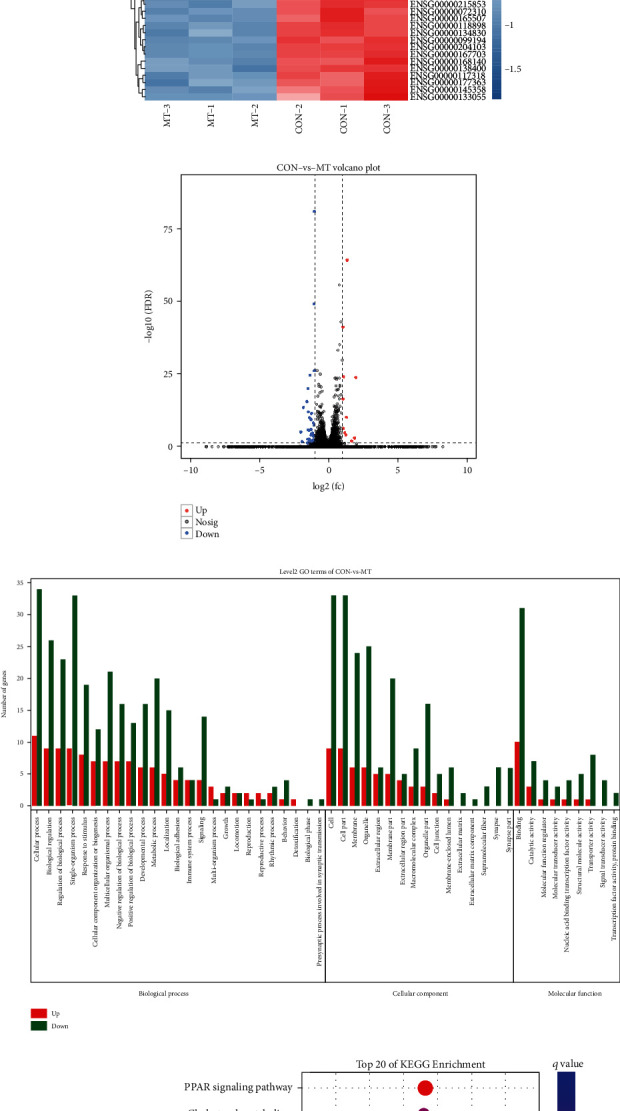
MT-induced differentially expressed genes. (a) Number of up- and downregulated genes. (b) Groups of the differentially expressed mRNAs: CON-1, CON-2, and CON-3 (0 nM MT); MT-1, MT-2, and MT-3 (150 nM). (c) Presentation of the volcano plot of DEGs identified from the RNA-seq. Red plots stand for upregulated genes, and blue plots indicate downregulated genes with the following criteria: *p* < 0.05 and absolute log_2_FC > 1. Grey plots indicate nonsignificantly expressed genes. The ordinate shows the -log10 of the adjusted *p* value for each gene, symbolizing the strength of the association. (d) GO analysis of the differentially expressed genes in cellular component, biological process and molecular function. (e) KEGG pathway analyzes the pathways involved in MT regulation.

To investigate the possible mechanisms of MT in inhibiting U87 glioma cells, we analyzed the transcription levels of associated mRNAs. Total RNAs were isolated from the control and MT-treated groups, and mRNA sequencing was performed. A total of 48 DEGs were obtained with a fold change ≥ 2 and *p* < 0.01. Among these, 37 genes were found to be upregulated, and 11 genes were downregulated in the MT-treated group compared with the control group (Figures 4(a)–4(c)). To determine the potential function of the DEGs, Gene Ontology (GO) enrichment analyses including categories of biological process (BP), cellular component (CC), and molecular function (MF) were performed. The results revealed that the DEGs were significantly enriched in GO0009987 (cellular process), GO0044464 (cell part), and GO0005623 (cell). GO0043226 (organelle), GO0016020 (membrane), GO0032501 (multicellular organismal process), GO0044425 (membrane part), GO0044422 (organelle part), GO0005576 (extracellular region), GO: 0031974 (membrane-enclosed lumen), GO0030054 (cell junction), and GO0044421 (extracellular region part) (Figure 4(d)).

The Kyoto Encyclopedia of Genes and Genomes (KEGG) pathway enrichment analysis revealed the top 20 significantly enriched pathways that were directly associated with MT. They involved peroxisome proliferator-activated receptor (PPAR), interleukin 17 (IL-17), adenosine 5′-monophosphate- (AMP-) activated protein kinase (AMPK), hematopoietic cell lineage, and cholesterol metabolism (Figure 4(e)).

### 3.4. PPI Network Analysis and Identification of Key Genes

**Figure 5:**
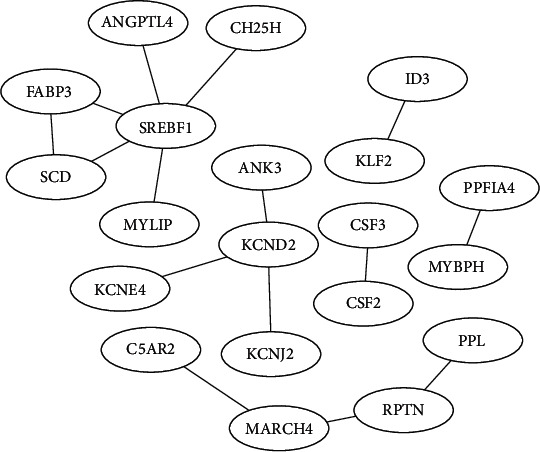
Results of the PPI. Each node represents a protein; each line is an interaction.

STRING was used to extract a PPI of the DEGs to evaluate potential interactions of the keg genes following 150 nM MT treatment for 12 h. A total of seven genes were identified from the PPI, including Sterol regulatory element-binding transcription factor 1 (SREBF1), membrane-associated ring-CH-type finger 4 (MARCH4), potassium voltage-gated channel subfamily E regulatory subunit 4 (KCNE4), angiopoietin-like 4 (ANGPTL4), myosin-binding protein H (MYBPH), periplakin (PPL), and complement C5a receptor 2 (C5AR2) (Figure 5). In addition, there are four more important genes protocadherin 18 (PCDH18), inhibitor of DNA-binding 3, HLH protein (ID3), DEPP1 autophagy regulator (DEPP1) and vasorin (VASN) reported in the literature. These genes mainly concentrated in the ion channels: membrane-related signaling pathways.

### 3.5. Relationship between the Key Genes and Survival Prognosis in Patients with Glioma

**Figure 6:**
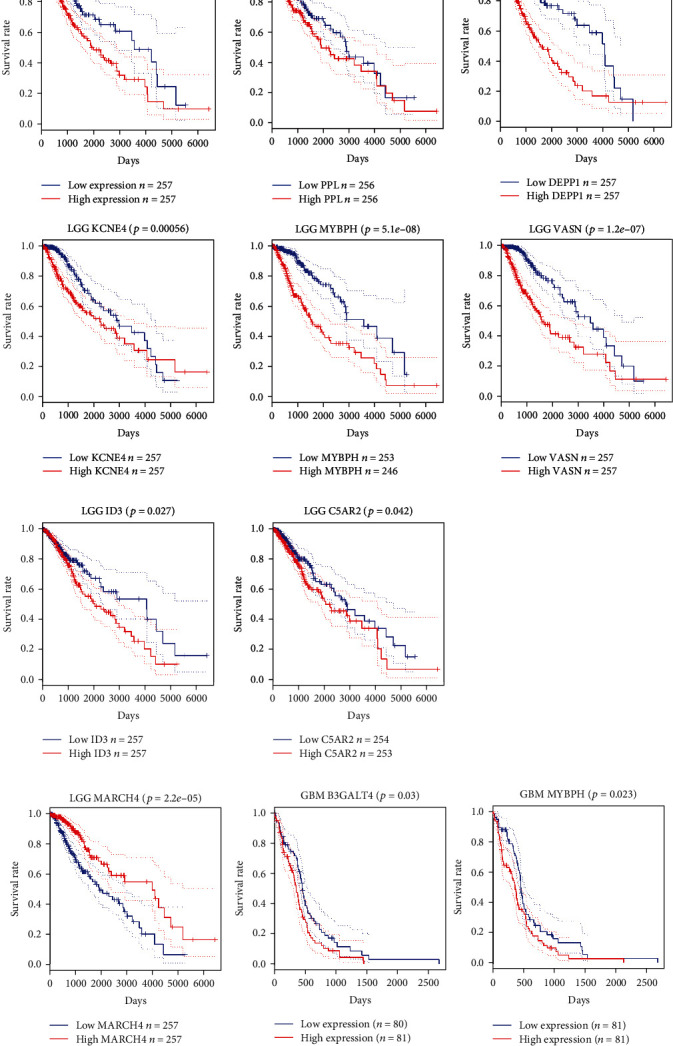
Kaplan-Meier analysis of overall survival (OS) in glioma patients with high and low expression of the key genes (red represents high expression; blue represents low expression). (a) The survival rate of patients with low expression of these genes were significantly higher than that of patients with high expression. Overexpression of PCDH18, PPL, DEPP1, VASN, KCNE4, MYBPH, C5AR2, and ID3 genes in patients with LGG was associated with poor survival. (b) Low expression of MARCH4 predicts poor survival in LGG patients. (c) Overexpression of B3GALT4 and MYBPH genes in GBM patients was associated with poor survival.

RNA-seq data and clinical data of glioma were obtained from TCGA database. Kaplan-Meier analysis was performed on the prognosis of patients with GBM and LGG in TCGA database, and survival curves were drawn. The survival curve revealed that in patients with LGG, there was a significant difference between the high-expression group and the low-expression group of PCDH18 (*p* < 0.001), PPL (*p* < 0.01), DEPP1 (*p* < 0.001), KCNE4 (*p* < 0.001), MYBPH (*p* < 0.001), VASN (*p* < 0.001), ID3 (*p* < 0.05), and C5AR2 (*p* < 0.05) (Figure 6(a)). Over a 15-year time horizon after diagnosis, the survival rate of patients with low expression of these genes were significantly higher than that of patients with high expression. These results suggested that overexpression of these genes in LGG patients were associated with poor survival. In addition, the survival rate of patients in LGG was significantly different between the low-expression group and the high-expression group of MARCH4 (*p* < 0.001) which suggested that low expression of the gene predicted poor survival (Figure 6(b)). In patients with GBM, B3GALT4 (*p* < 0.05) and MYBPH (*p* < 0.05) were significantly different between the high-expression group and the low-expression group. Overexpression of these genes were associated with poor survival rate in patients with GBM (Figure 6(c)). It is worth noting that MT administration reduced the expression levels of these genes in U87 cells compared with the control cells. These results indicated that the expression level of these genes in glioma is closely associated with the survival of patients, and may play an important role in the development of glioma. MT may promote the apoptosis of tumor cells by regulating the expression level of these genes.

### 3.6. Expression of Survival Key Genes in Grade 2 (G2) and Grade 3 (G3) Glioma Patients

**Figure 7:**
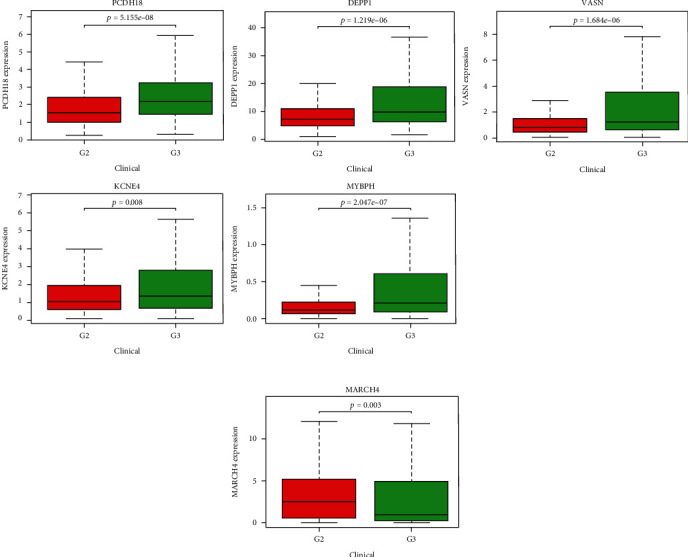
Comparison of key genes expression level in glioma grade 2 and grade 3 patients. (a) The expression levels of PCDH18, DEPP1, VASN, KCNE4, and MYBPH are higher in G3 than in G2. (b) The expression level of MARCH4 is lower at G3 than G2.

Of the 508 patients with glioma, the expression of PCDH18 (*p* < 0.001), DEPP1 (*p* < 0.001), VASN (*p* < 0.001), KCNE4 (*p* < 0.01), MYBPH (*p* < 0.001) and MARCH4 (*p* < 0.01) genes was significantly different between G2 and G3. Except that the expression of MARCH4 was decreased in G3, the expression of other genes was increased in G3 (Figures 7(a) and 7(b)).

### 3.7. Univariate Independent Prognostic Analysis

Univariate and multivariate Cox regression analyses were performed on 508 glioma patients to assess the influence of genes associated with glioma survival and other clinicopathological factors on survival status.

Univariate independent prognostic analysis was conducted to compare clinical traits and risk values individually with survival time and survival status to analyze whether these factors were associated with survival. Risk score *p* < 0.05 indicates that the correlation between the factor and survival is significant. Univariate independent prognostic analysis showed that age (*p* < 0.001), tumor grade (*p* < 0.001), PCDH18 (*p* < 0.05), PPL (*p* < 0.01), DEPP1 (*p* < 0.01), VASN (*p* < 0.001), KCNE4 (*p* < 0.001), MYBPH (*p* < 0.001), C5AR2 (*p* < 0.01), and MARCH4 (*p* < 0.01) gene expression levels were significantly different (Table 3). It suggested that age, grade, and the level of PCDH18, PPL, DEPP1, VASN, KCNE4, MYBPH, C5AR2, and MARCH4 genes were important predictors of survival.

### 3.8. Multivariate Factor Analysis

**Figure 8:**
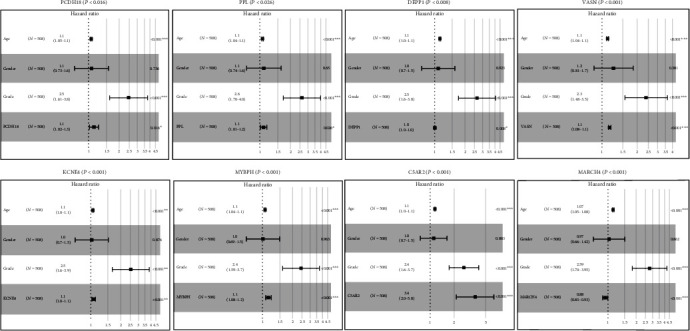
Multivariate Cox regression analysis was performed, and eight genes (PCDH18, PPL, DEPP1, VASN, KCNE4, MYBPH, C5AR2 and MARCH4) were selected to construct the risk signature.

Multivariate factor analysis is to compare clinical characteristics and risk values together with survival time and survival status, and it takes into account the influence of factors. Risk score *p* < 0.05 indicated that the risk value can be used as an independent prognostic factor [39]. Multivariate Cox regression analysis showed that the expression of PCDH18 (*p* < 0.05), PPL (*p* < 0.05), DEPP1 (*p* < 0.01), VASN (*p* < 0.001), KCNE4 (*p* < 0.001), MYBPH (*p* < 0.001), C5AR2 (*p* < 0.001), and MARCH4 (*p* < 0.001) was an independent predictor of glioma which is most closely related to the survival of patients with glioma, independent of other variables, and has significant prognostic value (Figure 8).

### 3.9. Quantitative Real-Time PCR Analyses of the Key Genes Related to MT Treatment

**Figure 9:**
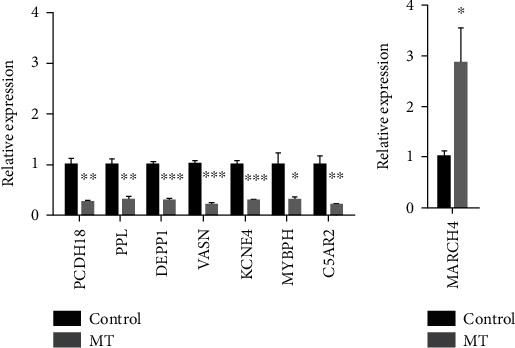
Relative expression of the key genes verified by qRT-PCR. (a) The results indicated that the expression of PCDH18, PPL, DEPP1, VASN, KCNE4, MYBPH, and C5AR2 in the MT group was lower in the MT-treated group than that in the control group. (b) The relative expression of MARCH4 in the MT group was higher than that in the control group. Results and expressed as the mean ± SEM, *n* = 3, ^∗^*p* < 0.05; ^∗∗^*p* < 0.01; ^∗∗∗^*p* < 0.001 vs. the control group.

In order to confirm the regulatory effect of MT on the eight key genes related to the prognosis of patients with glioma, the eight key genes between MT-treated (150 nM treatment for 12 h) and MT-untreated groups were selected and verified using qPCR. The gene expression levels, using the 2^−ΔΔCT^ method and normalization to GAPDH as a reference, were determined. The results showed that PCDH18, PPL, DEPP1, VASN, KCNE4, MYBPH, and C5AR2 genes were found to be downregulated (Figure 9(a)), and MARCH4 gene was upregulated compared to the control group (Figure 9(b)). This result suggested that MT can regulate the expression of these genes in glioma U87 cells. The expression profiles of these genes was consistent with the RNA sequencing data.

## 4. Discussion

In our study, we identified 48 genes that differed between MT treatment group and control group by transcriptome sequencing. GO analysis showed that these genes are mostly enriched in membrane-related signaling pathways such as membrane part, membrane, and membrane-enclosed lumen. The results suggest that MT mainly changed the cell morphology and destroyed cell membrane, thus inducing U87 cell death. KEGG enrichment analysis demonstrated that the signaling pathways associated with these DEGs were pathways in PPAR, IL-17, and AMPK signaling pathways. PPAR plays an important role in the occurrence and development of glioma, and PPAR agonists might represent novel adjuvant therapeutic agents for the treatment of gliomas [40]. In glioma samples, PPAR hyperactivation is associated with immunosuppression through increased regulatory T cell expression [41], and MT has been reported to be involved in T cell response and has immunomodulatory effects [42]. Whether MT could act as a PPAR agonist to promote tumor cell apoptosis by regulating the immune system will be explored in our future research.

Studies have shown that ion channel activity changes during tumorigenesis and development, and the abnormal expression and activity changes of ion channels are closely related to tumor cell proliferation and apoptosis [43]. In particular, the migration and invasion of glioma cells are promoted by ion channels and transporters [44]. Voltage-gated potassium channels are a large family of ion channels that are important transmembrane channels in neurons, regulating cell membrane potential and cell proliferation. Some studies have found that the occurrence and development of a variety of tumors are closely related to the dysregulation of voltage-gated potassium channel, such as glioma [45] and lung cancer [46]. The effects of voltage-gated potassium channels on proliferation and apoptosis of glioma cells have been widely studied, which provides a new therapeutic target for the prevention and treatment of glioma. In this study, KEGG enrichment analysis showed that voltage-gated potassium channels were abnormally expressed in U87 cells. It is noteworthy that MT may be involved in the apoptotic process of tumor cells by regulating voltage-gated potassium channels to change membrane function and influence membrane potential.

Through TCGA database, we identified that the eight key genes (PCDH18, PPL, DEPP1, VASN, KCNE4, MYBPH, C5AR2, and MARCH4) were associated with the survival of patients with glioma. Overexpression of PCDH18, PPL, DEPP1, VASN, KCNE4, MYBPH, and C5AR2 in glioma and low expression of MARCH4 were associated with poor survival. Interestingly, MT can downregulate the overexpression of PCDH18, PPL, DEPP1, VASN, KCNE4, MYBPH, and C5AR2 genes and upregulate the expression of MARCH4 gene in U87 cells. Independent prognostic analysis showed that eight genes are strongly associated with patient outcomes. It can be used as an independent prognostic factor in patients with glioma.

KCNE4 is expressed abundantly in the brain [47]. It can control the firing rate of neurons and synaptic transmission [48]. KCNE4 is one of the genes that modify ion channels in glioblastoma [49]. KCNE subunits are present in the immune system and may play a role in the immune system via associations with leukocyte K^+^ channels, specifically KCNE4, as novel targets for immunomodulation [50]. Our study showed that KCNE4 is closely associated with the prognosis of cancer patients. MT can inhibit the expression of KCNE4 gene on the gated potassium channel in U87 cells. MT may act as an inhibitor of potassium channel to inhibit the growth and proliferation of U87 cells and induce apoptosis of U87 cells. As a member of the ID protein family, ID3 plays an important role in cell proliferation, differentiation, and senescence and is involved in metastasis and angiogenesis of malignant tumors [51]. ID3 expression is increased in advanced glioma [52], and high ID3 expression had a shorter overall survival time in glioma patients [53]. Studies have shown that VASN protein has a cancer-promoting effect [54] and may be closely related to the occurrence and development of liver cancer [55]. Some studies found that glioma patients with high VASN expression had a shorter overall survival time. VASN stimulates tumor progression and angiogenesis in glioma and represents a novel therapeutic target for glioma [56]. Our findings were inconsistent with these studies. Importantly, we found that MT can downregulate the expression of VASN in U87 cells. MARCH4 plays an important regulatory role in the immune system, and its mechanism of action in glioma warrants further studied.

Although MT has the potential to inhibit tumor cell growth, maybe it is also cytotoxic to normal cells, limiting its clinical use [57, 58]. It has been suggested that this can be solved by targeted delivery of melittin nanoparticles [59]. Someone developed transdermal delivery preparations for bee venom, such as bee venom plastics with transdermal administration; it was found to be safe in the range of experimental doses and showed no acute or long-term toxicity [60]. In addition, the bee-sting therapy was used in clinical, and it was found that there were no abnormalities in blood, urine, stool routine, electrocardiogram, liver, and kidney function within the safe dose range [61]. Bee products and cupping are also commonly used in combination with bee-sting therapy, which effectively improve the quality of life and survival time of patients [62]. It was also reported that the toxicity of the peptide to normal cells was less [25], which may be related to the concentration of the peptide. Therefore, it is possible to reduce the toxicity of MT on normal tissues and cells through dose control or the use of drug delivery vectors.

## 5. Conclusion

It is worth noting that this study has certain limitations. For instance, no in vivo validation was performed, and an in-depth mechanistic exploration is needed. However, the current study provides important clues regarding that MT promotes apoptosis of U87 cells by regulating key genes that are mainly enriched in membrane potential, PPAR signaling pathway, and voltage-gated potassium channel. These genes are closely associated with the prognosis of patients with glioma. MT is a potential drug for the treatment of glioma.

## Figures and Tables

**Table 1:** qPCR primer sequences.

Gene	Primer sequence	Annealing T (°C)
PCDH18-F	GGAACAGAGGGTTGGATCAGT	59.4
PCDH18-R	GGCTCGAAATCGAACAGTAGAA	58.5
PPL-F	GGCTGCAGAATCTGGAGTTTGC	62.3
PPL-R	CTCAGTCTCCTCATCCAGTTCC	59.6
DEPP1-F	TGCCCACAATTCGGGAGAC	60.0
DEPP1-R	AGACCTCACGTAGTCATCCAG	59.0
VASN-F	TCTCACCTATCGCAACCTATCG	59.5
VASN-R	CAGACGGAGTAAGTGGCGTT	60.0
KCNE4-F	ACGATGAGCTGGAGGAGACCTC	63.2
KCNE4-R	CTCTAAGGTTGCTGGCTGATGG	61.0
MYBPH-F	TCCACATCCGAGAGAACATTGA	59.2
MYBPH-R	GAAGGGGATTTGCAGGTTGAC	59.5
C5AR2-F	CTGCTGACCATGTATGCCAG	58.7
C5AR2-R	CGCTGAACCGTAGACCACC	60.4
MARCH4-F	TTGGCTCATCTGGTCAACTTTC	58.9
MARCH4-R	GGTACACCGAGGGTCCTTCAT	61.2
GAPDH-F	GTCTCCTCTGACTTCAACAGCG	59.5
GAPDH-R	ACCACCCTGTTGCTGTAGCCAA	59.5

**Table 2:** Inhibitory concentration 50% (IC_50_) of MT.

IC_50_	U87	U251
24 h	178.07 ± 6.67 nM	185.20 ± 4.79 nM
48 h	125.71 ± 2.68 nM	151.59 ± 10.79 nM

**Table 3:** Univariate analysis of prognostic factors in the validation cohort.

Variables	Single-variate factor analysis						
	HR	95% CI	*p* value	Variables	HR	95% CI	*p* value
Age (years)	1.06	1.05-1.08	1.56*E*-15	VASN	1.10	1.08-1.12	6.66*E*-20
Gender	1.06	0.73-1.55	0.762	KCNE4	1.12	1.08-1.16	1.14*E*-09
Grade	3.12	2.06-4.72	7.44*E*-08	MYBPH	1.22	1.15-1.29	1.52*E*-11
PCDH18	1.13	1.03-1.24	0.013	C5AR2	2.61	1.45-4.71	0.001
PPL	1.11	1.04-1.20	0.003	MARCH4	0.91	0.85-0.96	0.002
DEPP1	1.01	1.00-1.01	0.004				

Abbreviations: HR: hazard ratio; CI: confidence interval.

## Data Availability

RNA-seq data and clinical data for glioma patients can be found in TCGA database (https://www.cancer.gov/tcga); the rest can be obtained from the corresponding author on reasonable request.

## Abbreviations

MT: Melittin
WHO: World Health Organization
CNS: Central nervous system
LGG: Low-grade glioma
HGG: High-grade glioma
GBM: Glioblastoma multiforme
TT Fields: Tumor-treating fields
RT: Radiotherapy
DEGs: Differentially expressed genes
RNA-seq: Transcriptome sequencing
PPI: Protein-protein interaction
TCGA: The Cancer Genome Atlas
TMZ: Temozolomide
CCK-8: Cell Counting Kit-8
GO: Gene Ontology
KEGG: Kyoto Encyclopedia of Genes and Genomes
SREBF1: Sterol regulatory element-binding transcription factor 1
KCNE4: Potassium voltage-gated channel subfamily E regulatory subunit 4
PCDH18: Protocadherin 18
PPL: Periplakin
DEPP1: DEPP1 autophagy regulator
MYBPH: Myosin-binding protein H
C5AR2: C5a anaphylatoxin chemotactic receptor 2
ID3: DNA-binding protein inhibitor ID-3
VASN: Vasorin
ANGPTL4: Angiopoietin-like 4
B3GALT4: Beta-1,3-galactosyltransferase 4
MARCH4: Membrane-associated ring-CH-type finger 4.
